# Audit of the first > 7500 noninvasive prenatal aneuploidy tests in a Swiss genetics center

**DOI:** 10.1007/s00404-021-06203-7

**Published:** 2021-09-17

**Authors:** Anahita Bajka, Michael Bajka, Fabian Chablais, Tilo Burkhardt

**Affiliations:** 1 Department of Obstetrics, University Hospital Zurich, Frauenklinikstr. 10, 8091 Zurich, Switzerland; 2 Department of Gynecology, University Hospital of Zurich, Zurich, Switzerland; 3 Genetica, Human Genetics and Genetic Counselling Unit, 8001 Zurich, Switzerland

**Keywords:** Cell-free DNA, SNP-based NIPT, Chromosomal aberration, Aneuploidy screening, Microdeletion

## Abstract

**Objectives:**

Noninvasive prenatal testing (NIPT) is actually the most accurate method of screening for fetal chromosomal aberration (FCA). We used pregnancy outcome record to evaluate a complete data set of single nucleotide polymorphism-based test results performed by a Swiss genetics center.

**Materials and methods:**

The Panorama^®^ test assesses the risk of fetal trisomies (21, 18 and 13), gonosomal aneuploidy (GAN), triploidy or vanishing twins (VTT) and five different microdeletions (MD). We evaluated all 7549 test results meeting legal and quality requirements taken in women with nondonor singleton pregnancies between April 2013 and September 2016 classifying them as high or low risk. Follow-up ended after 9 months, data collection 7 months later.

**Results:**

The Panorama^®^ test provided conclusive results in 96.1% of cases, detecting 153 FCA: T21 *n* = 76, T18 *n* = 19, T13 *n* = 15, GAN *n* = 19, VTT *n* = 13 and MD *n* = 11 (overall prevalence 2.0%). Pregnancy outcome record was available for 68.6% of conclusive laboratory results, including 2.0% high-risk cases. In this cohort the Panorama^®^ test exhibited 99.90% sensitivity for each trisomy; specificity was 99.90% for T21, 99.98% for T18 and 99.94% for T13. False positive rate was 0.10% for T21, 0.02% for T18 and 0.06% for T13.

**Conclusion:**

SNP-based testing by a Swiss genetics center confirms the expected accuracy of NIPT in FCA detection.

## Introduction

Introduced into clinical practice in late 2011 [1], fetal aneuploidy screening using the analysis of cell-free DNA (cfDNA) in maternal blood soon became confirmed as highly accurate and a method of choice for clinicians and pregnant women [1, 2]. Noninvasive prenatal testing (NIPT) set a new global standard in human genetics, changing the way in which prenatal medicine was perceived and practised, thereby also taking the ethical debate to new heights [3–6].

Different NIPT methods and tests are now available to clinicians [7, 8], including targeting or nontargeting methods [9, 10]. Each method offers its own advantages, disadvantages and analytical performance, which should be carefully assessed, validated and monitored by the scientific community and test providers. A critical aspect of the NIPT performance and quality has also been confirmed during the last years of experience with this analysis: the ratio of placental cfDNA to maternal cfDNA, known as the fetal fraction (FF), appears to correlate positively with gestational age and negatively with maternal body weight [7, 11–13].

This study reports on the Panorama^®^ test (Natera^®^ Inc., San Carlos, USA), a targeted SNP-based NIPT technique that screens for the most common fetal chromosomal aberrations (FCA), including trisomies (21, 18 and 13), gonosomal aneuploidy (GAN), triploidy or vanishing twins (VVT). Optionally, five microdeletion syndromes (MD) can be analyzed, in particular 22q11.2 deletion (DiGeorge) syndrome, 1p36 deletion syndrome, Prader–Willi syndrome, Angelman syndrome and 5p- (cri du chat syndrome) [14–16]. Following cfDNA isolation from the maternal plasma, amplification and sequencing, the data of each patient is independently analysed using the NATUS algorithm (Natera^®^) providing a precise risk score. A score ≥ 1/100 is considered as high risk and a score < 1/100 as low risk [11].

Our aim was to use pregnancy outcome record in an external evaluation of SNP-based NIPT results from a Swiss genetics center (Genetica AG, Zurich, Switzerland).

## Materials and methods

This was a retrospective analysis of FCA screening data prospectively collected in Switzerland. Between the introduction of NIPT analysis in April 2013 and September 2016 (3.5 years), Genetica AG collected 7549 test samples meeting legal and quality requirements from nondonor singleton pregnancies older than 9 weeks of gestation from duly briefed mothers at least 18 years of age who had signed written informed consent for further statistical test analysis.

The first 6159 samples (81.6%) in our study were analyzed by Natera^®^. From October 2015 onwards, the subsequent 1390 (18.4%) samples were analyzed in Zurich by Genetica AG following its approval as a Panorama^®^ test center. The optionally MD screening was available from July 2014.

Test results were classified as high or low FCA risk. As the Panorama^®^ test cannot differentiate between triploidy or a vanishing twin next to a normal fetus, the result needs checking by ultrasound and/or by an invasive diagnosis to be conclusive. All FCA were included. Also, since general health insurance does not cover NIPT on the basis of maternal age alone, reimbursement typically depends on an inconspicuous first trimester ultrasound including normal nuchal translucency and on finding a high risk in the first trimester test (cutoff value 1/1000) [17]. As a secondary outcome, the MDs were evaluated. The screening is a voluntary extension of NIPT and is always self-funded.

Genetica AG collected written outcome record from the referring clinicians and hospitals, if necessary, verbal outcome record was added. Follow-up closed 9 months after the last sample was taken (May 2017); outcome report collection ended 7 months later (December 2017) and was defined as aneuploidy positive or negative by invasive testing or clinical evaluation after delivery.

Genetica AG recorded and prepared all Panorama^®^ test results and outcome data for further evaluation; it also anonymized the data before release for external analysis.

Further analysis used Microsoft Excel 15.33 (Microsoft Corp., Redmond, USA), Stata 15.1 for Windows (StataCorp., College Station, USA) and R (R Core Team, Vienna, Austria).

The study was approved by the Zurich institutional review board (KEK-ZH-No.2016-00,672). It was undertaken for a master’s degree and was self-funded.

## Results

**Fig. 1:**
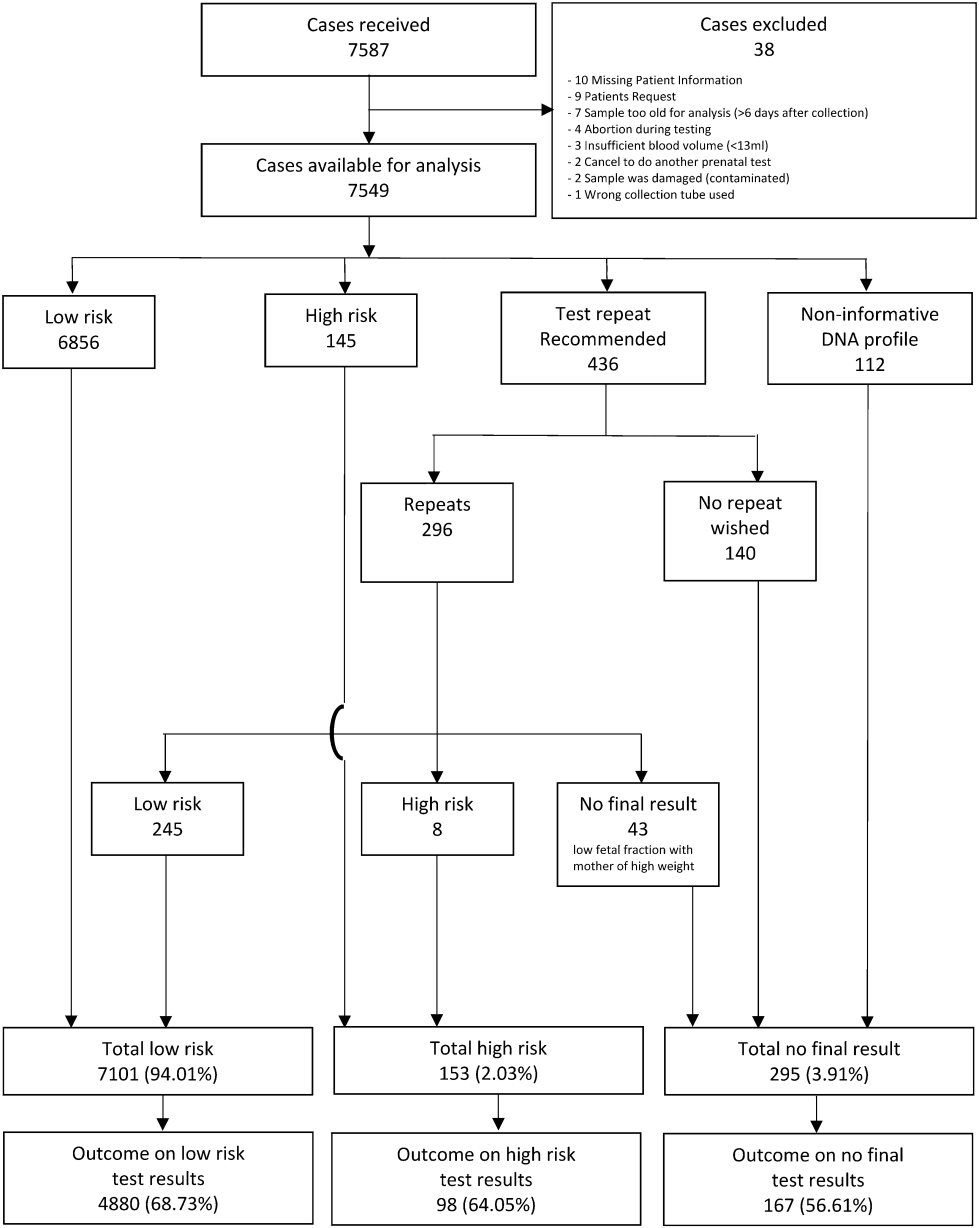
Study flow chart

**Fig. 2:**
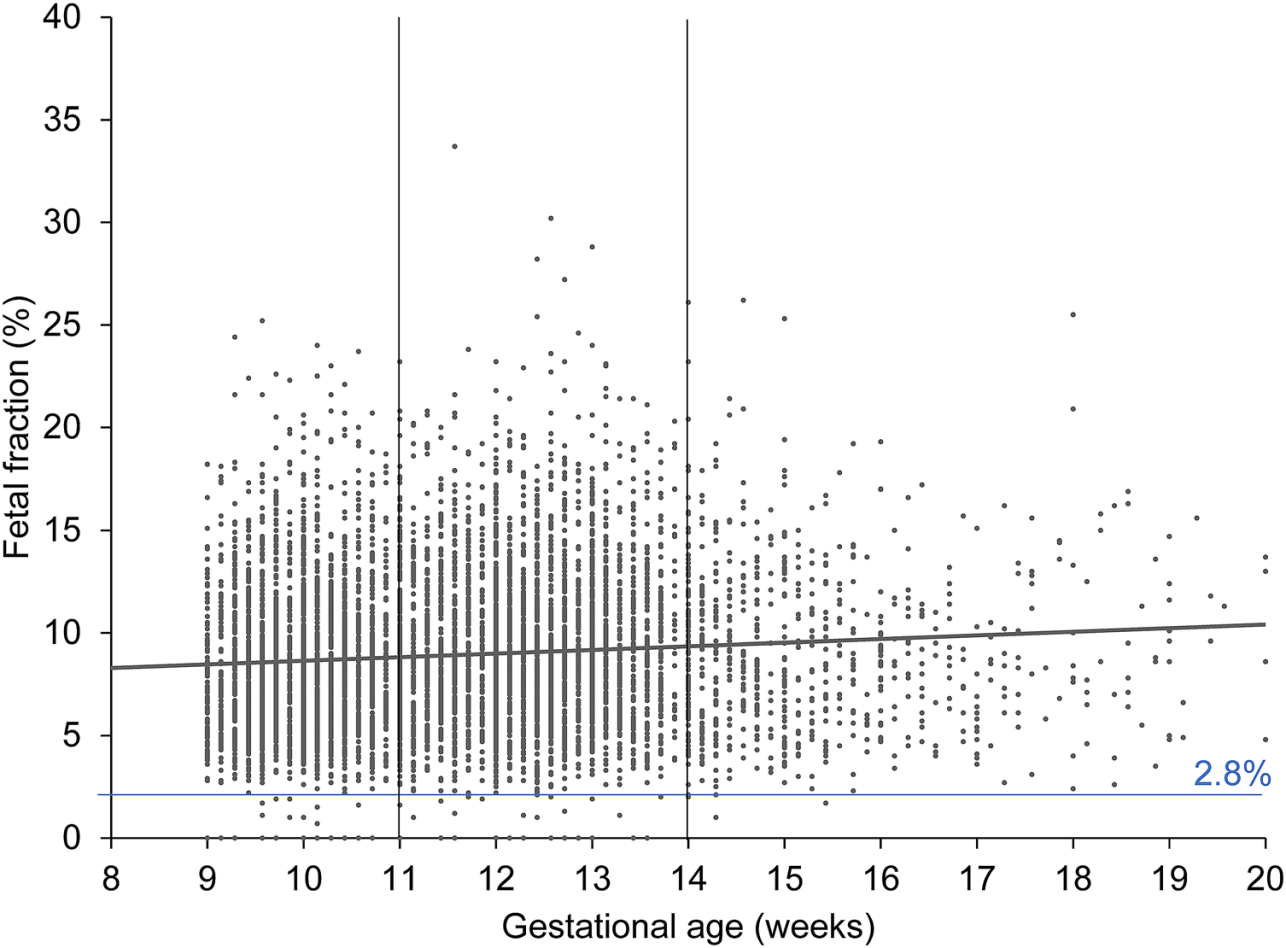
Relation between fetal DNA fraction of all cases and gestational age. In 108 pregnancies (1.4% (108/7549)) the FF was below 2.8%. The interval of 11 + 0 up until 14 + 0 weeks of gestation marked by two vertical lines

**Fig. 3:**
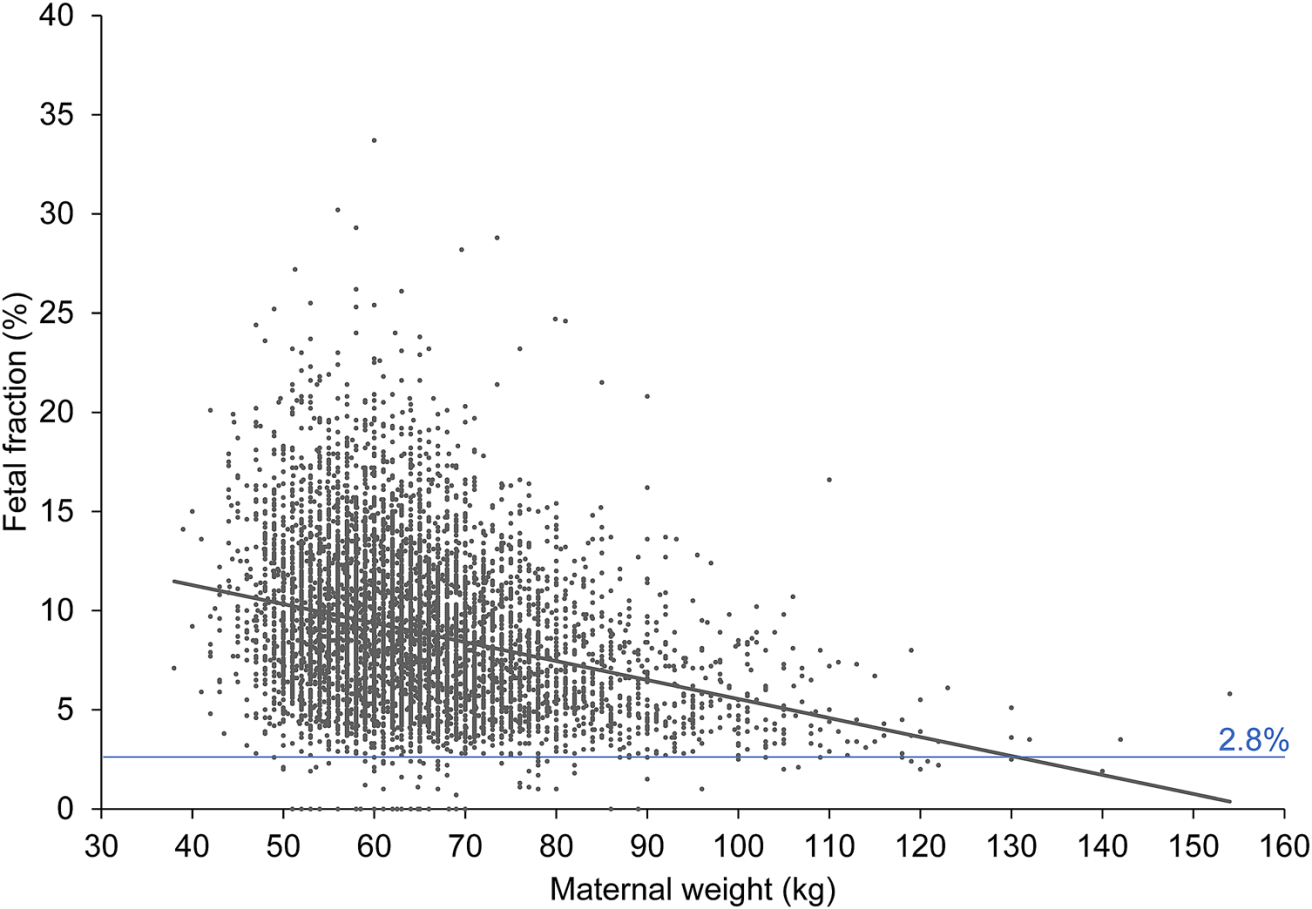
Relation between fetal DNA fraction of all cases and maternal weight

Of the 7587 samples received, 38 were excluded for various reasons (Fig. 1), leaving 7549 eligible for inclusion. Study population demographics (Table 1) showed that 3936 tests (52.1%) were performed between 11 + 0 (mean FF 9.1%) and 14 + 0 weeks of gestation (mean FF 9.4%), a range over which there is no significant correlation between FF and increasing gestational age. Between 9 and 20 weeks of gestation the positive correlation between fetal fraction and gestational age was significant (*p* < 0.001) (Fig. 2). However, FF decreased with increasing maternal weight (Fig. 3). In 108 pregnancies (1.4%, 108/7549) FF was below 2.8%. In 42 cases the fraction was below 2.8% even after repeat testing. According to the Swiss Society for Ultrasound, an inconspicuous so-called first-trimester ultrasound between 11 and 14 weeks of pregnancy is a prerequisite for performing a NIPT. 2906 tests (38.5%) were performed before 11 + 0 weeks, 36 tests after 20 + 0 weeks of gestation and two after 30 + 0 weeks.

**Table 1:** Demographics of the study population

Groups	Mean ± SD (range)
Mean maternal age [y]	35.5 ± 4.1 (18–56)
Mean maternal weight [kg]	62 ± 11.4 (38–154)
Mean gestational age [weeks + days]	11 + 5 ± 2 + 0 (9 + 0–34 + 6)

Regardless of the gestational age, the mean FF was 9.2% (SD 3.6) for the low-risk group, 8.3% (SD 4.6) for the high-risk group and 3.2% (SD 2.1) for the no result group. Table 2 lists test indications, mean maternal age and numbers of high-risk results. The mean interval between sample collection and test result was 9.2 days (range 3–20 days).

**Table 2:** Indications for testing

Indication	Cases [*n*] (%)	Maternal age [*n*]	High risk aneuploidy [*n*] (%)
Advanced maternal age	3898 (51.6)	38.0	76 (1.7)
On demand	2120 (28.1)	31.5	28 (1.3)
Noticeable first trimester screening	1302 (17.3)	34.8	47 (3.6)
Family history	94 (1.3)	35.4	1 (1.1)
Other**/**missing	135 (1.8)	32.8	1 (0.8)
Total	7549 (100)	35.5	153 (2.0)

Of the eligible 7549 test results, primarily 90.8% (6856/7549) were classified as low risk, 1.9% (145/7549) as high risk. Test repetition was recommended in 5.8% (436/7549) of the cases. In 1.5% (112/7549) of the cases, test repetition was not possible due to a noninformative SNP profile, being incompatible with the NATUS algorithm (Fig. 1). Of the cases with a recommended test repeat, the repetition gave a conclusive result in 85.5% of the cases. The remaining inconclusive cases were mainly caused by a low fetal fraction due to maternal obesity. The finally 153 high-risk results were as follows: T21 *n* = 76, T18 *n* = 19, T13 *n* = 15, GAN *n* = 19, VTT *n* = 13 and MD *n* = 11, overall prevalence 2.0% (153/7549). Once the MD screening was made available to the patients (from July 2014), MD screening was requested in 34.4% (1931/5618) of cases.

Pregnancy outcome record was available in 68.2% (5145/7549). Low-risk outcome in 4880 cases (in a total of 7101 low-risk cases) included no false negatives, giving a negative predictive value (NPV) of 100%.

The outcome data were available in 98 cases (64.1%) in the high-risk group. The high-risk result was evaluated in 4 cases with postnatal clinical evaluation (3 T21 results, 1 T13 result) an in 5 cases with genetic testing of material from miscarriage (2 T21 results, 1 T18 result, 1 GAN result). The outcome data in the high-risk group revealed 13 false positives: T21 *n* = 5, T18 *n* = 1, T13 *n* = 3, MD *n* = 3, GAN *n* = 1.

Some results lacked trisomy, VTT or GAN information. Thus, among the 606 incomplete low-risk cases, 11 lacked a T21 result, 13 a T18 result, 13 a T13 result, 23 a GAN result.

Tables 3 and 4 detail outcomes in the low risk, high risk and no result groups and show miscarriage/abortion rates. The miscarriage rate was higher in the high-risk group than in the low-risk group. The FCA rate was 6.6% in the no result group. Table 5 shows a comparison of results with and without outcome.

**Table 3:** Comparison of high-risk, low-risk and no final test results with outcome data

	Low risk test results		High risk test results		No final test results	
	Test result [*n*]	Outcome [*n*]	Test result [*n*]	Outcome [*n*]	Test result [*n*]	Outcome [*n*]
Trisomy 21	0	0	54	49	–	4
Trisomy 18	0	0	11	10	–	1
Trisomy 13	0	0	10	7	–	4
Microdeletions	0	0	8	5	–	1
Gonosomal aneuploidies	0	0	9	8	–	1
Triploidy or vanishing twin	0	0	6	6	–	0
Miscarriage**/**abortion rate	0.5%^a^ (22/4880)	0.5%^a^ (22/4880)	14.3%^a^ (14/98)	14.3%^a^ (14/98)	–	3.0%^a^ (5/167)
Normal	4880	4880	0	13	–	156
Total	4880	4880	98	98	167	167

^a^Miscarriage/abortion rate in percent

**Table 4:** Comparison of high-risk and low-risk test results without outcome

	Low risk test results [*n*]	High risk test results [*n*] (%)
Trisomy 21	0	22 (40)
Trisomy 18	0	8 (14.5)
Trisomy 13	0	5 (9.1)
Microdeletions	0	3 (5.5)
Gonosomal aneuploidies	0	10 (18.2)
Triploidy or vanishing twin	0	7 (12.7)
Normal	2221	0
Total	2221	55

**Table 5:** Comparison of test results with and without outcome

Groups	Outcome [*n*] (%)	No outcome [*n*] (%)
Low risk	4880 (94.9)	2221 (92.4)
High risk	98 (1.9)	55 (2.3)
Trisomy 21	54 (1.1)	22 (0.9)
Trisomy 18	11 (0.2)	8 (0.3)
Trisomy 13	10 (0.2)	5 (0.2)
Microdeletions	8 (0.2)	3 (0.1)
Gonosomal aneuploidies	9 (0.2)	10 (0.4)
Triploidy or vanishing twin	6 (0.1)	7 (0.3)
No result	167 (3.3)	128 (5.3)
Total	5145 (100)	2404 (100)

Evaluation of NIPT screening against pregnancy outcome yielded the following Panorama® test scores (Table 6): sensitivity 99.90% (95% CI 94.61–100%), specificity 99.73% (95% CI 99.53–99.85%), PPV 86.73% (95% CI 78.02–92.47%) and false positive rate (FPR) 0.27% (95% CI 0.15–0.47%). Sensitivity for each trisomy was 99.90% (95% CI for T21, 90.94–100%; 95% CI for T18, 65.54–100%; 95% CI for T13, 56.09–100%). Specificity was 99.90% (95% CI, 99.75–99.96%) for T21, 99.98% (95% CI 99.87–100%) for T18 and 99.94% (95% CI 56.09–100%) for T13; FPR was 0.1%, 0.02% and 0.06%, respectively. Sensitivity and specificity scores for GAN and VVT were similar. Sensitivity and specificity scores for MD were 99.9% (95% CI 63–100%) and 99.4% (95% CI 99.8–99.9%) respectively, PPV was 24.9% assuming a prevalence of 1: 4000.

**Table 6:** Sensitivity, specificity, FPR and PPV for all pathologic results with outcome

Groups	Sensitivity [%] (95% CI)	Specificity [%] (95% CI)	FPR [%] (95% CI)	PPV [%] (95% CI)
Trisomy 21	99.90 (90.94–100)	99.90 (99.75–99.96)	0.1 (0.037–0.254)	91.5 (96.4^a^) (87.93–96.54)
Trisomy 18	99.90 (65.54–100)	99.98 (99.87–100)	0.02 (0.001–0.130)	90.90 (57.12–99.52)
Trisomy 13	99.90 (56.09–100)	99.94 (56.09–100)	0.06 (0.015–0.200)	70.00 (83.3^a^) (35.37–91.90)
Microdeletions	99.90 (47.82–100)	99.4 (99.8–99.9)	37.5 (10.2–74.1)	24.9 (8.2–43.3)^b^
Gonosomal aneuploidies	99.90 (59.77–100)	99.98 (99.87–100)	0.02 (0.001–0.130)	88.89 (50.67–99.42)
Triploidy or vanishing twin	99.90 (51.68–100)	99.99 (99.99–100)	–	100 (51.68–100)

^a^PPV with consideration of a risk score cutoff value set at 90%

^b^Assuming a prevalence of 1:4000

## Discussion

The Sensitivity for trisomy 21, 18 and 13 was more than 99% with a high specificity of 99%. With regard to trisomy 21, 18 and 13, there was no false negative result. Comparing our trisomy detection results with those of meta-analyses and other studies, sensitivity and PPV were comparable [8].

FCA rates were typical of the distribution in an unselected population [2, 18, 19]. Comparisons of the groups with and without outcome data showed a similar distribution except for a slightly higher high-risk rate in the no-outcome record group (Table 5). The PPV for MD (24.9%), was similar to another study [16].

The observed test without conclusive results of 3.9% (295/7549) is also consistent with previous reports: a meta-analysis from 2017 found rates of test failure due to low FF ranging from 0.0 to 6.9%; there were no differences between methods of cfDNA analysis [7]. This number is remarkable as most nonrepeats (32.1%, 140/436) were down to patient refusal, not to the test itself. It is here described that one single repetition of the test will provide a result for 85.5% of the patients without a first conclusive result, which would theoretically reduce the final no-call rate from 3.9% down to 2.3% (175/7549). We therefore cannot confirm the higher rate of 6.4% reported for inconclusive results with this NIPT technique [20]. There are different reasons why a repetition of the test might be requested, knowing that only high-confidence results are reported. The cause can be a lower fetal fraction, when the cfDNA percentage is very low (less than 2.8%). Also, the DNA analyzed in some samples is inherently less informative (noisy data), making it difficult for the algorithm to obtain a high confidence result. These metrics include total cfDNA amount, number of reads and other control metrics that are in place to ensure good quality data for accurate, consistent results. In some situations, the DNA of a particular individual (mother or fetus) is not able to be interpreted. This can be due to missing pre-analytics information like multiple gestations or egg donor pregnancies for example. The other reasons for noninformative profiles can be vanishing twin pregnancies, fetal or maternal mosaicism or higher levels of homozygosity on the chromosomes tested (when the SNPs between mother and baby are too similar to yield informative results, possibly from consanguinity, segmental uniparental disomy or simply coincidence). The FCA rate in our no result group was 6.5%. After a nonconclusive result, the woman should be counseled about further genetic diagnostics.

The most common indications given for testing were maternal age and on demand (Table 2). Both are not accepted as an indication for insurance cover in Switzerland. GAN and MD screening are always self-funded, 34.4% of mothers requested MD testing. The maternal age as indication corresponds to the mean maternal age of 35.5 years. However, the mean maternal age in the Zurich population at the time was only 2 years lower (33.6 years) [21]. We can only speculate as to why 32.1% (140/436) of mothers declined a repeat after a test repeat was recommended, because repeating the test does not involve additional costs.

Most of the 36 tests done after 20 + 0 weeks of gestation were indicated by an abnormal second-trimester ultrasound scan; the remainder was on demand. These 36 cases are included in the analyses, but not shown in Fig. 2. According to the local and international guidelines, it is not recommended to perform an NIPT, if the first- or second-trimester ultrasound scan is abnormal [17, 22, 23]. We found no significant positive correlation between increasing FF and increasing gestational age, but we did identify a negative correlation between decreasing FF and increasing maternal weight (Fig. 3). This is in line with the results reported from other studies [7, 13, 20]. Each test provider specifies a lower limit for the FF. Natera^®^ defines FF values < 2.8% as a quality exclusion criterion, keeping in mind that 7.1% at a maternal weight of 100 kg and 51.1% at a maternal weight of 160 kg usually have a FF result below 4% [20, 24]. Leaving this threshold value aside, our analysis does suggest that FF in the high-risk group was slightly lower than in the low-risk group.

The weakest point in this retrospective analysis is no doubt the relatively low outcome record despite best efforts. This can at least be assumed to have impacted high- and low-risk cases equally. Outcome record was available in only 68.2% of cases, well below 90%, a value commonly exceeded in prospective studies [25]. The response rate greatly depended on the efforts of the institutions liaising between patients and Genetica AG. Ethical approval prevented intervention by a third party, before data collection and anonymization were complete.

In using a Panorama^®^ risk score, each patient receives an individual risk score based on the SNP test result, a few may be tested positive with a risk score < 99%. In these cases, the test result is more likely to be false positive. Of the false positive trisomy cases in our study, three out of five (T21) and one out of three (T13) had risk scores < 99%; thus, if we excluded test results with risk scores < 99%, the PPV for T21 in our study would be 96.4% instead of 91.5% and that for T13 would be 83.3% instead of 70.0%.

## Conclusion

Pregnancy outcome from 5145 of the 7549 mothers undergoing SNP-based testing by a Swiss genetics center between April 2013 and September 2016 confirms the expected accuracy of NIPT in FCA.
